# Potential Role for IL-2 ELISpot in Differentiating Recent and Remote Infection in Tuberculosis Contact Tracing

**DOI:** 10.1371/journal.pone.0011670

**Published:** 2010-07-20

**Authors:** Benjamin Krummel, Alan Strassburg, Martin Ernst, Norbert Reiling, Barbara Eker, Heidrun Rath, Robert Hoerster, Waltraud Wappler, Andrea Glaewe, Volker Schoellhorn, Giovanni Sotgiu, Christoph Lange

**Affiliations:** 1 Division of Clinical Infectious Diseases, Research Center Borstel, Borstel, Germany; 2 Division of Immune Cell Analytics, Research Center Borstel, Borstel, Germany; 3 Division of Microbial Interface Biology, Research Center Borstel, Borstel, Germany; 4 TB Surveillance, Public Health Department, Lübeck, Germany; 5 Autoimmun Diagnostika GmbH, Straßberg, Germany; 6 Hygiene and Preventive Medicine Institute, University of Sassari, Sassari, Italy; University of Stellenbosch, South Africa

## Abstract

Interferon (IFN)-γ release assays (IGRA) have improved tuberculosis contact tracing, but discrimination of recent from remote *Mycobacterium tuberculosis* contacts is not possible by IGRA alone. We present results of a tuberculosis contact investigation with a new early-secretory-antigenic-target (ESAT)-6 and culture-filtrate-protein (CFP)-10 specific interleukin (IL)-2 ELISpot in addition to ESAT-6 and CFP-10 specific IFN-γ ELISpot and tuberculin skin testing (TST). Results of the TST, IFN-γ ELISpot and IL-2 ELISpot were positive in 6/172 (3.4%), 7/167 (4.2%) and 6/196 (3.1%) of contacts, respectively. Close contact (≥100 hours) to the index case increased the risk of positive results in the IFN-γ ELISpot, TST, and IL-2 ELISpot by 40.8, 19.3, and 2.5 times, respectively. Individuals with a positive IFN-γ ELISpot/negative IL-2 ELISpot result had a median (IQR) duration of index case exposure of 568 hours (133_1000) compared to individuals with a positive IFN-γ ELISpot/positive IL-2 ELISpot result (median = 24 hours; 20_130; p-value = 0.047). Combination of a *M. tuberculosis* specific IFN-γ ELISpot with a *M. tuberculosis* specific IL-2 ELISpot significantly improved the identification of individuals with the highest risk of recent *M. tuberculosis* infection and is a promising method that should be explored to target tuberculosis preventive chemotherapy.

## Introduction

Identification and treatment of individuals with lasting *Mycobacterium tuberculosis* specific immune responses (latent tuberculosis infection -, LTBI) following recent contact to a contagious index case with pulmonary tuberculosis are crucial to tuberculosis control [1]. The diagnosis and management of LTBI has evolved over the last decade with improvements in the immunodiagnosis by *M. tuberculosis* specific interferon-γ release assays (IGRA) in addition to the tuberculin skin test [2]. In principle, IGRA detect interferon-γ release by mononuclear cells in the ELISpot (T-SPOT.*TB* – Oxford Immunotec, Abingdon, UK) or in whole blood in the ELISA (QuantiFERON-TB GOLD in tube; QFT-G-IT; Cellestis, Carnegie, Australia) following *ex vivo* contact with antigens that are naturally encoded in the *M. tuberculosis* genomic region of difference (RD)-1 and RD11 (only QFT-G-IT). The genes encoding for these proteins are not present in most non tuberculous mycobacteria and are deleted from the genome of *M. bovis* Bacille Calmette Guerin (BCG) strains that are used for tuberculosis vaccinations [3], [4]. The population of T lymphocytes responding rapidly with the production of IFN-γ following *ex vivo* stimulation with these antigens belongs predominantly to the pool of CD4+ CD45RA-CCR7- effector memory cells that likely had recent antigen exposure [5]. In contrast, long-lived central memory T-cells may persist even after successful treatment of tuberculosis [6], [7] and are less likely to release IFN-γ after short incubation with antigen while they are more likely to produce IL-2 upon antigen exposure than effector cells [7]. Thus, assaying *M. tuberculosis*-specific T cell function defined solely by the quantification of IFN-γ secretion after short term *ex vivo* incubation with *M. tuberculosis*-specific antigen may prove to be an insufficient indicator for recent inhalation exposure to *M. tuberculosis*. In contrast, simultaneous measures of other T cell function, such as *M. tuberculosis*-specific stimulation of IL-2 secretion may improve the discrimination of active versus remote LTBI [7].

A 28-year-old schoolteacher was referred to our hospital for diagnostic evaluations for presumptive tuberculosis. Between March and October 2007 he had consulted seven different general practitioners complaining of progressive cough and weight loss until finally in November 2007 a chest x-ray was performed and a cavitating lesion in the left upper lobe of the lung was demonstrated.

In our hospital, alcohol acid fast bacilli (AAFB) were readily identified on sputum smears. The bacilli were identified as *M. tuberculosis* by culture and susceptibility testing revealed antibiotic drug resistance to isoniacid and streptomycin. We estimated by the presence of his symptoms that he had been infectious for a period of seven months prior to hospital admission. During this period, he had exposure to a large number of close contacts including pupils and teachers from two different schools and members of a church parish.

We describe the results of tuberculosis contact tracing among well-defined contacts of a highly symptomatic sputum smear positive index case, comparing a novel *M. tuberculosis* antigen specific IL-2 ELISpot assay with a *M. tuberculosis* antigen specific IFN-γ ELISpot and the TST for the diagnosis of LTBI. This outbreak provided an opportunity to test the hypothesis that simultaneous measures of *M. tuberculosis*-specific IFN-γ and IL-2 secretion may improve identification of individuals with the highest risk of recent infection with *M. tuberculosis*.

## Materials and Methods

### Study population

Individuals with close contact to the symptomatic index case during the period of March 2007 until October 2007 were identified by the municipal health care department of the city of Luebeck, Germany.

Contacts were asked to estimate their exposure time to the index case in hours per week. We identified a group of close (with a total exposure of 100 hours or more) and another of occasional contacts (exposure of less than 100 hours). For 76 contacts who did not provide any information about exposure to the index case, the time was estimated according to the average exposure of classmates. In addition information about BCG vaccination status, possible former contact to tuberculosis, and pre-existing illness and medication were obtained. Data were recorded on a standardized questionnaire.

All adults and children who were enrolled agreed by written informed consent to participate in the study. In addition, the agreement of both legal guardians was obtained for the children who were enrolled. The study was approved by the ethical committee of the University of Luebeck, Germany.

### Tuberculin skin test (TST)

Tuberculin skin testing was performed by intracutaneous injection of 2U/0.1ml of tuberculin RT23 (Statens Serum Institut, Copenhagen, Denmark) on the volar aspect of the forearm. The TST was administered and read by experienced staff from the Municipal Health Authority. Indurations were measured at 48–72 hours by two independent readers [8]. Parallel to TST testing blood was taken from contacts after informed consent following an eight week period after the last exposure to the index case [9]. Chest radiographs of the TST-positive contacts were performed, and clinical and radiographic findings were reviewed for evidence of active tuberculosis.

### ELISpot assay

ELISpot assays for IFN-γ and separately for IL-2 release by mononuclear cells were performed using test-plates from AID, Strassberg, Germany. Briefly, 250,000 PBMCs were plated overnight on 96-well plates, which had been pre-coated with a mouse anti-human IFN-γ or IL-2 antibody, in 200 µl culture medium per well. The cells were left unstimulated (negative control), were stimulated with 10 ng/ml anti-CD3 monoclonal antibody (clone X35, Beckman-Coulter, Krefeld, Germany; positive control) and with 5 µg/ml of ESAT-6 and CFP-10 peptides (kindly provided by AID, Strassberg, Germany). Culture of the plates, washing, counterstaining, visualisation and analysis of the spots was performed according to the manufacturer guidelines.

The response of stimulated cultures was considered positive when the test well contained >5 spots and had at least twice the number of spots than the negative control well. The background number of spots in negative control wells was always less than five spots per well. The response of stimulated cultures was considered negative when the test well contained ≤5 spots or less than twice the number of spots in the unstimulated control well and when the positive control well contained at least 50 spots at the same time.

### Analysis of host activating potential of the index cases *M. tuberculosis* strain

Human THP1 cells were differentiated for 96h in the presence of 5 ng/ml PMA. Human monocyte-derived macrophages (MDM) were generated as previously described [10]. *Mycobacterium tuberculosis* strains H37Rv (ATCC 27294) and the index cases *M. tuberculosis* strain (9729/07) were grown at 37°C in Middlebrook 7H9 broth supplemented with Middlebrook OADC-Enrichment (BD Biosciences) to an OD of 0.2–0.4. Cultures were harvested, aliquoted, and frozen at −80°C. Viable cell counts were determined by plating serial dilutions of cultures on Middlebrook 7H10 agar plates (BD Biosciences). For experimental in vitro infections, aliquots were diluted in culture media and the preparation was passed six times through a 27-gauge needle to ensure proper dispersion of mycobacteria.

THP-1 (3×10^5^/ml) cells and MDM (5×10^5^) cells were stimulated in 500 µl cultures for 4h in the absence or presence of Mtb strains H37Rv or 9729/07 at the indicated multiplicity of infection (MOI). Bacterial lipopolysaccharide (LPS) of *Salmonella enterica*, serotype friedenau H909, was kindly provided by Prof. Dr. H. Brade (Research Center Borstel, Borstel, Germany) and used as positive control. Supernatants were frozen at −80°C until analysis. The formation of TNF-α was analyzed by ELISA (TNF Duoset, R&D Systems), as recommended by the manufacturer.

### Statistical analysis

Data were analysed using Stata 9.0 (StataCorp, Stata Statistical Software Release 9, College Station, TX, USA, 2005). Categorical data were compared using the Chi-squared test (or Fisher's exact test, when expected cell sizes were smaller than five). The Wilcoxon-Mann-Whitney test was used for continuous measurements to test relationships in unpaired analysis, when assumed that the dependent variable is a not normally distributed interval variable. Since there is no gold standard for the determination of LTBI, the analysis was focussed on estimating the strength of association between the degree of exposure (the duration and proximity of contact with the infectious case) and the test results. Logistic regression analysis was performed in order to evaluate which covariates have had an association with a positive test result. Test concordance was assessed by Cohen's κ-statistics with agreement considered slight’for k≤0.2, ‘fair’ for 0.2<k≤0.4, ‘moderate’for 0.4<k≤0.6, ‘substantial’for 0.6<k≤0.8 and ‘optimal’for 0.8<k≤1.0. A Spearman correlation was used in order to evaluate the relationship between two variables (contacts' hours of exposure with IFN-γ or IL-2 ELISpot test results, respectively), assumed to be not normally distributed and interval. The tests were performed as two-sided tests. Test results were regarded to be statistical significant if the p-value was <0.05.

## Results

**Figure 1:**
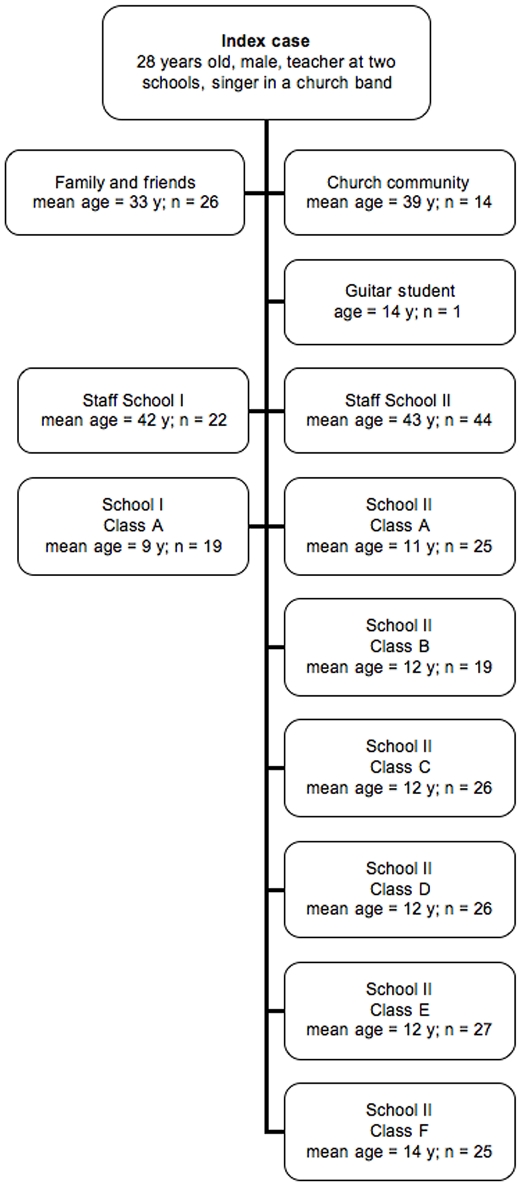
Pattern of exposure among 274 contacts of a teacher with sputum acid fast bacilli smear-positive pulmonary tuberculosis.

Among the 274 close contacts were the index patient's family and close friends, members of his church community including the church music group, where he participated as guitarist, colleagues and staff from the two schools where he worked as a teacher, students from seven different school classes, and a guitar student. The patterns of exposure are shown in **figure 1**.

### Tuberculin skin test

A total of 172 (62.7%) of 274 contacts were tested with the TST. Only six (3.4%) out of 172 developed a TST induration of ≥5 mm which is regarded as a positive test result in recent contacts according to national guidelines in Germany [9].

Eighteen of 172 (10.5%) contacts reported a history of BCG-vaccination. Three of these 18 individuals (16.7%) had a positive TST result. Seventy-four (43.0%) individuals reported that they had not been vaccinated with BCG. In this group, 1/74 (1.4%) had a positive TST result. In the remaining 80 individuals, mostly school children, the BCG vaccination status was unclear. In this group 2/80 (2.5%) had a positive TST result.

### ELISpot assays

**Figure 2:**
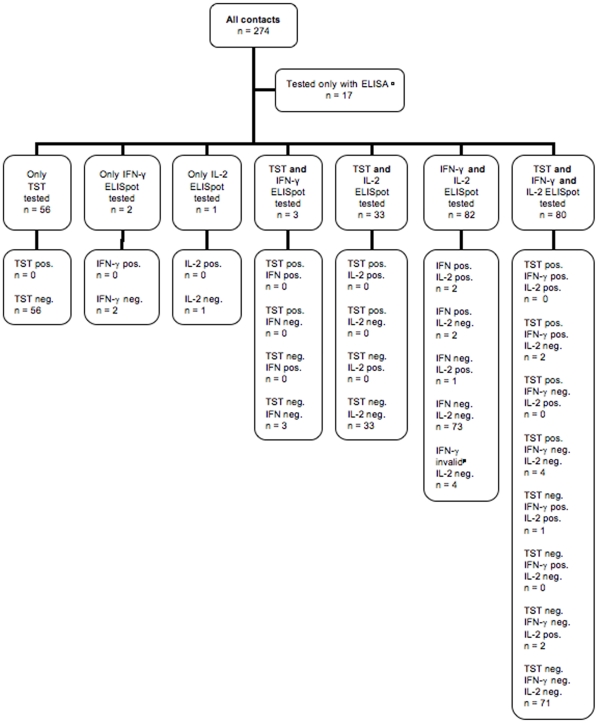
Distribution of immunodiagnostic test results. TST: tuberculin skin test; ELISpot: enzyme-linked immunospot. ^Δ^ In these 17 cases a skin test could not be performed. Because we did not obtain their informed consent for participation, these individuals were not tested with ELISpot in the study but tested by the city's health authorities with ELISA (*QuantiFERON-TB Gold*). $ These 2 contacts did not receive IL-2 ELISpot testing because they had only agreed on testing with test methods already approved by the health authorities. We performed IFN-γ ELISpot here. *§* In this case the amount of blood taken from the student was not sufficient to perform both ELISpot tests. This contact later received ELISA testing from the city's health authorities, in which she showed a negative result. * These 3 contacts or their parents did not agree on IL-2 ELISpot testing but only on test methods already approved by the health authorities. *^#^* These 4 contacts showed an indeterminate IFN-γ ELISpot result. Because skin testing could not be performed, the city's health authorities then tested these 4 with ELISA, in which all of them showed a negative result.

One hundred ninety-six of 274 (71.5%) were tested with IL-2 ELISpot while only 167/274 (60.9%) contacts were tested with IFN-γ ELISpot (**figure 2**).

In 34 cases the volume of blood sampled was insufficient to perform both assays. Since for our study IL-2 ELISpot testing was more interesting we generally performed IL-2 readout first when the amount of blood was insufficient for both assays. In 33 of 34 cases we also performed additional skin testing, one contact later received ELISA testing by the city's health authorities (*QuantiFERON-TB Gold*). All these 34 cases were negative on IL-2 testing and also on additional TST or ELISA, respectively.

In five other cases we performed IFN-γ ELISpot without IL-2 readout, three of these contacts were also tested with TST (**figure 2**). In these five cases the contacts or their parents had only agreed on testing with methods already approved by the German national guidelines. All five contacts showed negative IFN-γ ELISpot results, the TST were also negative.

### IL-2 ELISpot assay

In 6/196 (3.1%) the test IL-2 ELISpot assay result was positive. Among these six contacts three had been positive on IFN-γ ELISpot testing while three had been negative. The positive test results are summarized in **table 1**.

**Table 1:** Contacts with positive result in at least one of the immunodiagnostic tests.

Contact number	TST result	IFN-γ ELISpot result	IL-2 ELISpot result	BCG vaccination	Duration of exposure (total hours)	Age (years)	Country of birth	Former TB contact
51	**pos.**	neg.	neg.	yes	60	35	Germany	no
224	**pos.**	neg.	neg.	not known	56	12	unknown	not known
247	**pos.**	neg.	neg.	yes	72	31	Germany	no
255	**pos.**	neg.	neg.	no	12	39	Germany	no
276	**pos.**	**pos.**	neg.	yes	1000	56	Germany	no
282	**pos.**	**pos.**	neg.	not known	136	32	Germany	yes
261	not tested	**pos.**	neg.	no	130	43	Germany	no
278	not tested	**pos.**	neg.	yes	1000	58	Germany	no
127	not tested	**pos.**	**pos.**	not known	24	51	Germany	yes
258	not tested	**pos.**	**pos.**	yes	130	39	Germany	no
216	neg.	**pos.**	**pos.**	no	20	13	Germany	no
252	neg.	neg.	**pos.**	no	32	15	Germany	no
43	neg.	neg.	**pos.**	not known	48	45	Germany	no
85	not tested	neg.	**pos.**	no	32	14	Germany	no

TST: Tuberculin skin test; ELISpot: enzyme-linked immunospot; BCG: Bacille Calmette-Guérin.

Among the six contacts tested positive on IL-2 ELISpot was a 51 year-old female teacher who reported intensive tuberculosis contact more than 20 years ago. She did not receive antituberculosis preventive chemotherapy despite having a positive TST reaction at that time. In her case the net numbers of the IL-2 specific spots (11 in the CFP-10 ELISpot, 10 with ESAT-6) were highest among all contacts. Her IFN-γ ELISpot result was also positive. A TST had not been performed, and her vaccination status was unknown. This woman's exposure to the index case was quantified as 24 contact hours.

Among those three contacts tested positive on IL-2 ELISpot while being negative on IFN-γ ELISpot were two contacts, a student and a teacher, with negative TST results. One had not been vaccinated; the other's BCG status was unknown. Their time of exposure was 32 and 48 hours, respectively. The third one, a 14-year-old boy, had not received skin testing. He had not been vaccinated. His time of exposure was 32 hours.

### IFN-γ ELISpot assay

In 7/167 (4.2%), the test results with the IFN-γ ELISpot were positive. In 156 cases, the IFN-γ ELISpot test results were negative, and in four cases (2.4%) the test results were indeterminate. In these 4 contacts TST could not be performed. In such cases the city's health authorities performed mandatory ELISA testing (QFT-G-IT). All four individuals showed a negative test result in the QFT-G-IT. The distribution of test results is shown in **figure 2**.

All six TST-positive contacts were also tested by IFN-γ ELISpot. Two (33.3%) of them had a positive IFN-γ ELISpot result, four (66.7%) showed a negative IFN-γ ELISpot result. The positive test results are summarized in **table 1**.

### Influence of exposure time on test results

The median, Inter-Quartile Range –IQR-, time of exposure to the index case was much higher among the contacts that were positive on IFN-γ ELISpot testing (130, 24_1000, hours) than among those positive on IL-2 ELISpot (32, 24_48, hours), but the difference was not statistically significant (p-value = 0.15).

For TST positive contacts the median (IQR) time of exposure was 66 (56 _136) hours.

No statistically significant difference was found when the median time of exposure among the contacts who were notified as TST positive was compared to that of those with a positive IFN-γ result (p-value = 0.52) or a positive IL-2 ELISpot result (p-value = 0.15).

**Figure 3:**
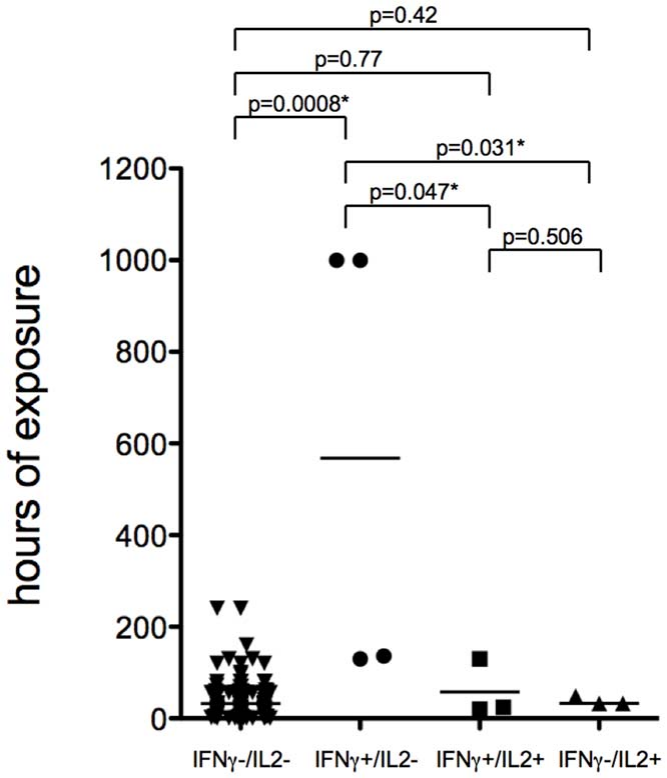
Results of *M. tuberculosis* specific enzyme linked immunospot (ELISpot), assaying interferon (IFN)- γ and interleukin (IL)-2 production in response to *ex vivo* contact of peripheral blood mononuclear cells with early antigenic target (ESAT)-6 and culture filtrate protein (CFP)-10 in relation to the cumulative time of exposure to an infectious index case. * Differences are statistically significant.

Individuals with a positive IFN-γ results and a negative IL-2 ELISpot result had a median (IQR) duration of exposure to the index case of 568 hours (133_1000) compared to individuals with a positive IFN-γ results and a positive IL-2 ELISpot result with a median (IQR) duration of exposure to the index case of 24 hours (20_130; p-value = 0.047) or individuals with a negative IFN-γ results and a positive IL-2 ELISpot result with a median (IQR) duration of exposure to the index case of 32 hours (32_48; p-value = 0.031) (**figure 3**).

Logistic regression analysis of the association of a positive test (IL-2 ELISpot assay, IFN-γ IGRA and TST) with potential explanatory covariates for diagnosis of LTBI was performed (**Table 2**). Only when the independent variable “close contact” (i.e. exposure time ≥100 hours) was associated to the positive result of IFN-γ IGRA, highly statistically significant odds ratios were obtained on both the univariate (40.8, 95%CI 6.9_240.3; p-value<0.0001) and multivariate analysis (27.9, 95%CI 4.2_183.5; p-value = 0.001); other statistically significant ORs obtained in the univariate analysis and associated to covariates, like age with TST, age with IFN-γ IGRA and close contact with TST, were not confirmed by a multi-variable model (**table 2**).

**Table 2:** Logistic regression analysis of the association of a positive interleukin (IL)-2 ELISpot assay, interferon (INF)-γ ELISpot assay and tuberculin skin test with potential explanatory factors for the presence of positive results in tests for the immunodiagnosis of tuberculosis.

Covariates	IL-2 ELISpot assay		INF-γ ELISpot assay		Tuberculin skin test	
	*Crude OR* *(95% CI)*	*Adjusted OR* *(95% CI)*	*Crude OR* *(95% CI)*	*Adjusted OR* *(95% CI)*	*Crude OR* *(95% CI)*	*Adjusted OR* *(95% CI)*
*Exposure ≥100 hours*	2.5(0.3 23.0)p<0.001)	1.7(0.2 18.0)p = 0.6	40.8(6.9 240.3)p<0.001	27.9(4.2 183.5)p = 0.001	19.3(2.7 137.5)p = 0.003	8.5(0.9 81.6)p = 0.06
*BCG vaccination*	1.4(0.5 3.4)p = 0.51	1.2(0.4 3.2)p = 0.72	1.7(0.7 4.0)p = 0.21	1.36(0.4 4.4)p = 0.6	2.3(0.9 5.9)p = 0.07)	2.3(0.7 7.3)p = 0.15
*Age, years*	1.02(1.0 1.1)p = 0.3	1.02(1.0 1.1)p = 0.44	1.06(1.0 1.1)p = 0.008)	1.06(1.0 1.13)p = 0.07	1.05(1.0 1.1)p = 0.02	1.05(1.0 1.1)p = 0.05)
*Sex, male*	0.8(0.1 4.5)p = 0.8	0.8(0.1 4.5)p = 0.8	1.1(0.2 5.1)p = 0.9	0.8(0.1 4.8)p = 0.72	1.05(0.3 2.5)p = 0.29	0.37(0.1 4.05)p = 0.41

*OR, odds ratio; CI, confidence interval.; BCG, Bacillus Calmette-Guérin.

Agreement between TST and IFN-γ ELISpot results and between IFN-γ ELISpot and IL2 ELISpot results was moderate (kappa = 0.42 and 0.44, respectively) while it was poor between TST and IL2 ELISpot results (**table 3**).

**Table 3:** Inter reliability of diagnostic methods for the diagnosis of lasting immune responses to *M. tuberculosis*.

Diagnostic test		Diagnostic test			Cohen's kappa (SE)	Agreement*
		IFN-γ release ELISpot				
		*Positive*	*Negative*	*Total*		
**TST**	*Positive*	2	4	6	0.42(0.1)	*Moderate*
	*Negative*	1	76	77		
	*Total*	3	80	83		

		Tuberculin Skin Test				
		*Positive*	*Negative*	*Total*		
**IL-2 release ELISpot**	*Positive*	0	3	3	−0.04(0.1)	*Less than chance*
	*Negative*	6	104	110		
	*Total*	6	107	113		

		IFN-γ release ELISpot				
		*Positive*	*Negative*	*Total*		
**IL-2 release ELISpot**	*Positive*	3	3	6	0.44(0.1)	*Moderate*
	*Negative*	4	148	152		
	*Total*	7	151	158		

*Criteria based on the interpretation of Landis and Koch are used.

### Genotyping and analysis of TNF formation by the *M. tuberculosis* strain of the index case *ex vivo*

The *M. tuberculosis* strain of the index case and the laboratory strain H37Rv were genotyped as described previously [11]. MIRU-VNTR identified the strain of the index case to be of an East African Indian genotype, whereas the laboratory reference strain H37Rv was typed to belong to the Haarlem subtype. The cytokine release of macrophages and dendritic cells to infection with a given *M. tuberculosis* isolate is critical to induce a successful induction of the antimycobacterial response of the host.

**Figure 4:**
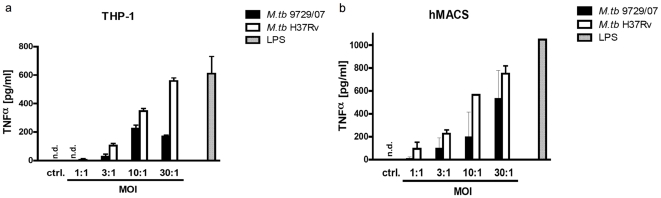
TNF-α production of A) human THP-1 cells and of B) human monocyte-derived macrophages (MDM) in response to the M. tuberculosis strain of the index case (9729/07) and the laboratory strain H37Rv. PMA-differentiated human THP-1 cells and MDM were incubated with indicated multiplicities of infection (MOI) of viable *M. tuberculosis*. Supernatants were harvested 24 h after infection and TNF levels were determined by ELISA. Data from one representative experiment of three are shown (LPS: lipopolysacharide; ctrl.: medium control, n.d.: not detectable).

We studied the TNF release of human differentiated THP-1 cells and human monocyte-derived macrophages in response to the *M. tuberculosis* strain of the index case and compared it to the laboratory strain H37Rv. In **figure 4** it is shown that the *M. tuberculosis* strain of the index case lead to a 30–50% reduced TNF formation compared to the lab strain H37Rv.

## Discussion

In this tuberculosis contact investigation with a highly symptomatic index case we performed TST and ELISpot testing with both IFN-γ and IL2 readouts among a large group of asymptomatic close contacts.

Three important results emerged from this study: 1.) Despite a high level of exposure among a large group of contacts, less than 5% had a positive IGRA response post exposure indicating possible *M. tuberculosis* infection. None of the contacts developed active tuberculosis. 2.) *Ex vivo* infection of monocytes and macrophages with the *M. tuberculosis* strain of the index case showed a significant reduction in the capacity to induce TNF when compared to the laboratory strain H37Rv, related to the attenuated virulence of this strain. 3.) Despite the low number of individuals with LTBI in this cohort, the combination of IFN-γ and IL2 ELISpot allowed for a significantly better discrimination of the level of exposure to the index case than IGRA alone.

With the introduction of IGRAs important advances have been achieved in the immunodiagnosis of LTBI [12] and active tuberculosis [13], [14]. However, when IGRA are performed on cells from the peripheral blood alone, they cannot discriminate individuals with active tuberculosis from those with LTBI [15]. In addition, in individuals with LTBI, IGRAs are not able to identify those with recently acquired infection, who have the highest risk of progression to active tuberculosis [16], [17].

A potential method to improve the ability of IGRA to identify individuals with recently acquired LTBI is a *M. tuberculosis* specific IL2 release assay in addition to the IGRA. Interleukin-2 is promoting T cell replication and is essential for cellular immunity and granuloma formation. While IFN-γ is predominantly produced by effector memory T cells, IL-2 is predominantly produced by central memory T cells. Recently, a shift in the IFN-γ and IL-2 cytokine profile from a codominance of IFN-γ-only and IFN-γ/IL-2-secreting T cells and the appearance of IL-2-only secreting T-cells during and after treatment of tuberculosis has been demonstrated [18].

With increasing level of exposure to the index case, the chance of a positive IFN-γ ELISpot test increased by more than 40 times while the risk of a positive IL-2 ELISpot increased only by 2.5 times in this study. Individuals with a positive IFN-γ ELISpot result and a negative IL-2 ELISpot result had the highest cumulative duration of exposure to the index case and therefore the highest chance of recent contact to *M. tuberculosis*. In contrast, a positive result of the *M. tuberculosis* specific IL-2 ELISpot was not significantly related to a higher exposure to the index case. In agreement with previous longitudinal observations of IFN-γ and IL2 specific immune responses in patients with active tuberculosis undergoing antituberculosis treatment [7] the additional IL-2 ELISpot did significantly improve the discrimination of putative recent from remote infection with *M. tuberculosis* in our cohort.

Airborne transmission of *M. tuberculosis* is more likely when duration and proximity of contact with an infectious case increase [19], [20]. The minimum amount of exposure that results in infection and the time of exposure that makes *M. tuberculosis* transmission inevitable remain unknown. In our study the index case had probably been infectious for a period of seven months. However, none of the contacts developed active tuberculosis. This is a lower rate compared to other school outbreaks (e.g. in [21], where in a group of 535 contacts nine secondary cases occurred out of 97 cases with LTBI).

Well-documented cases of infection resulting from brief exposure have been published [22]. Ewer et al. found in a British school outbreak [21] “…that 130 hours sharing room air with a person with sputum smear positive cavitatory tuberculosis is certain to result in infection”. However, in our study we found that among those tested negative with all three test methods were also contacts with up to 240hours of exposure, suggesting that the degree of airborne *M. tuberculosis* transmission and/or the infectiousness of patients with sputum AFB smear-positive cavitatory tuberculosis can be very variable.

Despite the fact that the index case needed to be considered to be highly contagious by the presence of large numbers of acid fast bacilli on sputum smears we observed a positive IGRA response post exposure in less than 5% of close contacts indicating possible *M. tuberculosis* infection. To evaluate the possibility of an attenuated virulence of this strain of M. tuberculosis, we consequently compared the cytokine release following infection of human THP-1 cells and monocyte derived macrophages with this strain and an H37Rv reference strain of *M. tuberculosis*.

Genetic diversity of *M. tuberculosis* isolates may have implications for the outcome of the disease. In a murine model of experimental tuberculosis infection it was reported that genetically different *M. tuberculosis* strains evoked markedly different immunopathological events [23]. Bacteria with the Beijing genotype, highly prevalent in Asia and Southern Africa, elicited a non-protective immune response in mice and were the most virulent. The cytokine Tumor necrosis factor (TNF) is essential for the development of protective immunity against *M. tuberculosis* infection [24]. Granuloma formation and granuloma maintenance are critically dependant on the presence of TNF [25], [26]. This has been demonstrated in different experimental settings, including anti-TNF antibody treatment, TNFRp55 deficiency and TNF-deficiency (reviewed in [27]). Systemically reduced TNF levels, e.g. in the presence of TNF targeted biologicals have been shown to significantly enhance the risk of reactivation of tuberculosis [28], [29], [30] in latently infected individuals. However in *in vitro* settings the role of TNF may be more complex than previously expected in particular when human immune cells are analyzed: We found a significant reduction in the ability to induce TNF in human monocytes and macrophages following *ex vivo* infection with the *M. tuberculosis* strain of the index case, when compared to the virulent laboratory strain H37Rv. Of interest in a similar study it has been shown that infection of human alveolar macrophages with virulent *M. tuberculosis* strains *in vitro* induced the secretion of significantly higher levels of bioactive TNF than attenuated strains correlating with their ability to multiply intracellularly [31]. In addition treatment of infected human macrophages with neutralizing anti-TNF antibodies reduced the growth rate of intracellular bacteria, whereas bacterial replication was augmented by addition of exogenous TNF [31]. Similar results have also been obtained with human monocytes [32]. Tumor necrosis factor promotes growth of virulent *Mycobacterium tuberculosis* in human monocytes iron-mediated growth suppression is correlated with decreased release of TNF from iron-treated infected monocytes]and also human dendritic cells [33]. Inverse correlation of maturity and antibacterial activity in human dendritic cells. Based on these findings, one is tempted to speculate that the low TNF release by the strain of the index case may be related to a reduced virulence and a relatively low transmission rate of this particular strain among contacts. However this analysis was merely exploratory. A more detailed analysis integrating a wide array of clinical isolates needs to be done in order to accurately type the strain with respect to strain-specific immune responses.

Several limitations of our study need to be addressed. As this was an *ad hoc* outbreak evaluation with a predominant population of children, not all contacts participated in the study and not all tests for the immunodiagnosis of tuberculosis could be performed in all contacts in parallel. Also, because of the relative low frequency of positive IGRA responses among close contacts, conclusions about exposure time and test results as well as the added value of IL-2 ELISpot cannot be definitive and need to be confirmed in similar outbreaks with a higher number of individuals with positive IGRA responses. Due to low number of cases possibilities of statistical processing of TST positive and IFN-γ and IL-2 ELISpot negative cases are limited.

This current study provides no conclusive evidence, but contains some intriguing observations that, following *ex vivo* contact with *M. tuberculosis* specific antigens, recently acquainted infection with *M. tuberculosis* is related to a positive IFN-γ ELISpot and a negative IL-2 ELISpot while remote infection with *M. tuberculosis* is related to a positive IFN-γ ELISpot and a positive IL-2 ELISpot. Longitudinal studies with larger numbers of contacts with positive M. tuberculosis-specific immune responses will be needed for a further exploration and clarification of this hypothesis. Finally, a causative relation between the observed *ex vivo* augmentation of TNF induction in monocytes and macrophages and the *in vivo* reduced transmission rate of this strain of *M. tuberculosis* has not been established.

### Conclusion

Despite intense contact to a highly symptomatic AFB-smear positive index case, transmission of *M. tuberculosis* only rarely occurred in this tuberculosis outbreak possibly related to attenuated virulence of this strain.

Combination of a *M. tuberculosis* specific IFN-γ ELISpot with a *M. tuberculosis* specific IL-2 ELISpot improved the identification of individuals with the highest risk of recent *M. tuberculosis* infection and is a promising tool for the discrimination of recent from remote LTBI that should be further explored.

## References

[1] Mack U, Migliori GB, Sester M, Rieder HL, Ehlers S (2009). LTBI: latent tuberculosis infection or lasting immune responses to M. tuberculosis? A TBNET consensus statement.. Eur Respir J.

[2] Nienhaus A, Schablon A, Diel R (2008). Interferon-gamma release assay for the diagnosis of latent TB infection–analysis of discordant results, when compared to the tuberculin skin test.. PLoS ONE.

[3] Mahairas GG, Sabo PJ, Hickey MJ, Singh DC, Stover CK (1996). Molecular analysis of genetic differences between Mycobacterium bovis BCG and virulent M. bovis.. J Bacteriol.

[4] Gey van Pittius NC, Sampson SL, Lee H, Kim Y, van Helden PD (2006). Evolution and expansion of the Mycobacterium tuberculosis PE and PPE multigene families and their association with the duplication of the ESAT-6 (esx) gene cluster regions.. BMC Evol Biol.

[5] Streitz M, Tesfa L, Yildirim V, Yahyazadeh A, Ulrichs T (2007). Loss of receptor on tuberculin-reactive T-cells marks active pulmonary tuberculosis.. PLoS ONE.

[6] Vincenti D, Carrara S, De Mori P, Pucillo LP, Petrosillo N (2003). Identification of early secretory antigen target-6 epitopes for the immunodiagnosis of active tuberculosis.. Mol Med.

[7] Millington KA, Innes JA, Hackforth S, Hinks TS, Deeks JJ (2007). Dynamic Relationship between IFN-{gamma} and IL-2 Profile of Mycobacterium tuberculosis-Specific T Cells and Antigen Load.. J Immunol.

[8] Sokal JE (1975). Editorial: Measurement of delayed skin-test responses.. N Engl J Med.

[9] Diel R, Forssbohm M, Loytved G, Haas W, Hauer B (2007). [Recommendations for background studies in tuberculosis].. Pneumologie.

[10] Reiling N, Blumenthal A, Flad HD, Ernst M, Ehlers S (2001). Mycobacteria-induced TNF-alpha and IL-10 formation by human macrophages is differentially regulated at the level of mitogen-activated protein kinase activity.. J Immunol.

[11] Wirth T, Hildebrand F, Allix-Beguec C, Wolbeling F, Kubica T (2008). Origin, spread and demography of the Mycobacterium tuberculosis complex.. PLoS Pathog.

[12] Diel R, Loddenkemper R, Meywald-Walter K, Niemann S, Nienhaus A (2008). Predictive value of a whole blood IFN-gamma assay for the development of active tuberculosis disease after recent infection with Mycobacterium tuberculosis.. Am J Respir Crit Care Med.

[13] Jafari C, Ernst M, Kalsdorf B, Greinert U, Diel R (2006). Rapid diagnosis of smear-negative tuberculosis by bronchoalveolar lavage enzyme-linked immunospot.. Am J Respir Crit Care Med.

[14] Jafari C, Thijsen S, Sotgiu G, Goletti D, Dominguez Benitez JA (2009). Bronchoalveolar Lavage Enzyme-linked Immunospot for a Rapid Diagnosis of Tuberculosis: A TBNET Study.. Am J Respir Crit Care Med.

[15] Goletti D, Stefania C, Butera O, Amicosante M, Ernst M (2008). Accuracy of immunodiagnostic tests for active tuberculosis using single and combined results: a multicenter TBNET-Study.. PLoS ONE.

[16] Hart PD, Sutherland I (1977). BCG and vole bacillus vaccines in the prevention of tuberculosis in adolescence and early adult life.. Br Med J.

[17] IUTLD (1982). Efficacy of various durations of isoniazid preventive therapy for tuberculosis: five years of follow-up in the IUAT trial. International Union Against Tuberculosis Committee on Prophylaxis.. Bull World Health Organ.

[18] Millington KA, Innes JA, Hackforth S, Hinks TS, Deeks JJ (2007). Dynamic relationship between IFN-gamma and IL-2 profile of Mycobacterium tuberculosis-specific T cells and antigen load.. J Immunol.

[19] Grzybowski S, Barnett GD, Styblo K (1975). Contacts of cases of active pulmonary tuberculosis.. Bull Int Union Tuberc.

[20] Kenyon TA, Valway SE, Ihle WW, Onorato IM, Castro KG (1996). Transmission of multidrug-resistant Mycobacterium tuberculosis during a long airplane flight.. N Engl J Med.

[21] Ewer K, Deeks J, Alvarez L, Bryant G, Waller S (2003). Comparison of T-cell-based assay with tuberculin skin test for diagnosis of Mycobacterium tuberculosis infection in a school tuberculosis outbreak.. Lancet.

[22] Small PM, Hopewell PC, Singh SP, Paz A, Parsonnet J (1994). The epidemiology of tuberculosis in San Francisco. A population-based study using conventional and molecular methods.. N Engl J Med.

[23] Lopez B, Aguilar D, Orozco H, Burger M, Espitia C (2003). A marked difference in pathogenesis and immune response induced by different Mycobacterium tuberculosis genotypes.. Clin Exp Immunol.

[24] Flynn JL, Goldstein MM, Chan J, Triebold KJ, Pfeffer K (1995). Tumor necrosis factor-alpha is required in the protective immune response against Mycobacterium tuberculosis in mice.. Immunity.

[25] Egen JG, Rothfuchs AG, Feng CG, Winter N, Sher A (2008). Macrophage and T cell dynamics during the development and disintegration of mycobacterial granulomas.. Immunity.

[26] Algood HM, Lin PL, Flynn JL (2005). Tumor necrosis factor and chemokine interactions in the formation and maintenance of granulomas in tuberculosis.. Clin Infect Dis.

[27] Stenger S (2005). Immunological control of tuberculosis: role of tumour necrosis factor and more.. Ann Rheum Dis.

[28] Keane J, Gershon S, Wise RP, Mirabile-Levens E, Kasznica J (2001). Tuberculosis associated with infliximab, a tumor necrosis factor alpha-neutralizing agent.. N Engl J Med.

[29] Gomez-Reino JJ, Carmona L, Valverde VR, Mola EM, Montero MD (2003). Treatment of rheumatoid arthritis with tumor necrosis factor inhibitors may predispose to significant increase in tuberculosis risk: a multicenter active-surveillance report.. Arthritis Rheum.

[30] Wolfe F, Michaud K, Anderson J, Urbansky K (2004). Tuberculosis infection in patients with rheumatoid arthritis and the effect of infliximab therapy.. Arthritis Rheum.

[31] Engele M, Stossel E, Castiglione K, Schwerdtner N, Wagner M (2002). Induction of TNF in human alveolar macrophages as a potential evasion mechanism of virulent Mycobacterium tuberculosis.. J Immunol.

[32] Byrd TF (1997). Tumor necrosis factor alpha (TNFalpha) promotes growth of virulent Mycobacterium tuberculosis in human monocytes iron-mediated growth suppression is correlated with decreased release of TNFalpha from iron-treated infected monocytes.. J Clin Invest.

[33] Buettner M, Meinken C, Bastian M, Bhat R, Stossel E (2005). Inverse correlation of maturity and antibacterial activity in human dendritic cells.. J Immunol.

